# Assessing parental marital quality and divorce related to youth sexual experiences and adverse reproductive health outcomes among 50,000 Chinese college students

**DOI:** 10.1186/s12978-022-01531-6

**Published:** 2022-12-01

**Authors:** Wen Wang, Ruoyu Yin, Wenzhen Cao, Yu Wang, Tingkai Zhang, Yan Yan, Kun Tang

**Affiliations:** 1 Vanke School of Public Health, Tsinghua University, No. 30 Shuangqing Road, Haidian District, Beijing, 100084 China; 2 Lee Kong Chian School of Medicine, Nanyang Technological University, 11 Mandalay Road, Singapore, 308232 Singapore; 3 School of Public Health and Management, Wenzhou Medical University, Chashan University Town, Wenzhou, 325035 Zhejiang China; 4 Fudan University Law School, No. 2005 Song Hu Road, Yangpu District, Shanghai, 200438 China; 5 School of Life Sciences, Tsinghua University, No. 30 Shuangqing Road, Haidian District, Beijing, 100084 China; 6 The Fitting Piece, 68 Redstone Road, Suffield, CT 06078 USA

**Keywords:** Parental marital quality, Divorce, Parental marital status, Sexual behaviors, College students

## Abstract

**Background:**

This study aimed to investigate the associations between parental marital quality, divorce, and sexual and reproductive health outcomes among Chinese young people.

**Methods:**

The study included 51,124 students from a large-scale cross-sectional study in China from 2019 to 2020. The exposures were parental marital quality and legal marital status reported by students. The dichotomous outcomes included sexual experiences, high-risk sexual behaviors, unintended health outcomes, and sexual abuse. Multivariate logistic regression models adjusting for socio-demographic factors were used to assess the relationship between parental marital quality, divorce, and sexual and reproductive health outcomes, stratified by sex.

**Results:**

A total of 10.72% of the surveyed students’ parents had divorced. Participants from divorced family rated perceived parental marital quality less than half of the ratings on a 10-point scale of those from intact family (3.22 vs. 7.44). Parental divorce was associated with a higher likelihood of sexual abuse, high-risk sexual behaviors, and unintended health outcomes. A higher perceived parental marital quality was associated with a lower probability of adverse sexual and reproductive health experiences and outcomes, such as forced penetrative vaginal or anal intercourse (male: OR: 0.73, 95% CI: 0.64–0.83; female: OR:0.71, 95% CI: 0.65–0.77), casual sexual intercourse (male: OR: 0.78, 95% CI: 0.73–0.83; female: OR: 0.77, 95% CI: 0.72–0.83), and sexually transmitted infections (male: OR: 0.79, 95% CI: 0.70–0.89; female: OR: 0.82, 95% CI: 0.73–0.91).

**Conclusions:**

Parental marital quality and status are associated with poorer sexual and reproductive health outcomes among young adults, suggesting that specific intervention programs should be implemented for children from unharmonious families or divorced families to prevent adverse sexual and reproductive health outcomes.

## Plain Englishsummary

Marital relationships in China have undergone significant transformations with the rapid industrialization and modernization of the society. The prevalence of divorce in China and worldwide has shown an upward trend in recent years. Divorce and parental marital quality often affect youth health, but its association with youth sexual and reproductive health requires more discussion. This study aimed to investigate the associations between parental marital quality and parental divorce status (marriage or divorce) and a range of youth sexual and reproductive health indicators. Participants included 51,124 students from universities or vocational colleges across all 31 provinces in mainland China. A total of 10.72% of the surveyed students’ parents had divorced. Participants from divorced family rated perceived parental marital quality less than half of the ratings on a 10-point scale of those from intact family (3.22 vs. 7.44). Divorce and lower parental marital quality were separately associated with a higher likelihood of experiencing sexual abuse, high-risk sexual behaviors, and unintended health outcomes among youth. These findings highlight the importance of implementing specific intervention programs to protect children from unharmonious families or divorced families.

## Introduction

Sexual behaviors, especially high-risk sexual behaviors (HRSB), which are associated with an increased risk of sexually transmitted infections (STI) and unintended pregnancy, are critical health and social issues [1]. Youth is an essential transition from adolescence to sexually mature adulthood when people begin to form individual sexual values and have sexual activities. College students in China have become increasingly liberal in sexual attitudes, such as being more active in sexual behaviors and having multiple sexual partners, because of rapid modernization and an open-minded social culture [2]. However, access to sex education is still largely limited, which could be reflected in the increasing trend of risky sexual behaviors among college students. A recent survey of teenagers in 11 provinces in China reports that 26.6% of sexually active college students have casual sex, which could lead to sexual risk-taking such as unprotected sex [3]. Therefore, it is critical to investigate the potential factors associated with sexual and reproductive health (SRH) in Chinese youth.

Children in single-parent families or with poor parental marital quality, including parental conflicts, a precursor to parent divorce, were reported to have increased sexual activities [4–8] and more frequent HRSB in adolescence [9–15]. Specifically, an unharmonious parental marital relationship was correlated with a higher risk of sexual debut before 18 years old, unprotected sex, and multiple sexual partners in adolescence [16–20]. Moreover, living with both parents is associated with delaying the onset of being sexually active in young people in some lower-to-middle-income countries [10, 21]. However, whether the associations differ across males and females requires more investigation.

According to the United Nations Demographic Yearbook 2011, the divorce rate increased substantially worldwide, and especially in China, the divorce rate doubled from 2002 to 2010 [22]. China’s crude divorce rate has unprecedentedly exceeded 3‰ every year since 2016 [23] and this trend is expected to continue [24]. It could be attributable to the increasing influence of economic growth, urbanization, and employment becoming in China over the years [25]. Although previous studies reported the association between divorce and adverse sexual behaviors in adolescents [7, 26–28], the potential effects of the timing of parental divorce on children’s sexual behaviors are largely unknown.

Most current studies focus on western countries or Africa, while research in Asia settings is scant. To our knowledge, no previous study has examined the relationship between parental marital status and adolescent sexual abuse in the Chinese context, and only few studies investigate the impact of parental marital status on risky sexual behaviors or adverse health outcomes. Therefore, the purpose of the current study was to explore the relationships between perceived parental marital quality, parental divorce, and sexual experiences and adverse reproductive health outcomes among university and vocational college students in China. Specifically, the study aimed to: (i) explore the association between parental marital status and SRH experiences, behaviors, and adverse outcomes in youth in China; (ii) investigate whether the association differs by the timing of parents’ divorce; (iii) examine the association between perceived parental marital quality and SRH experiences, behaviors, and adverse outcomes, and; (iv) assess whether the above associations differ across females and males.

## Methods

### Study design and participants

Data were derived from a large-scale cross-sectional study of social media-based National College Student Survey on Sexual and Reproductive Health (NCSS-SRH), which comprehensively measured youth SRH and related factors in China from November 2019 to February 2020. The study was commissioned by China Family Planning Association (CFPA) and collected by China Youth Network (CYN).

Higher education institution (HEI) study sites were selected using a multi-stage sampling technique considering school locations (eastern, central, or western regions), school types (vocational college, or university), grades, and sex, under which the probability of a university or a vocational college being selected was proportional to the number of students reported in the Educational Statistics Yearbook of China 2018 [29]. An anonymous self-administered questionnaire was distributed to students via the two most widely used social media platforms (WeChat and Sina Weibo) snowballing through the official societies or associations of CYN under each HEI site. To avoid repetitive responses, we used cookie-based duplication protection to automatically restrict each device from submitting the questionnaire more than once.

A total of 55,757 participants responded and submitted the questionnaire. Inclusion criteria for eligible participants in our analyses were (1) who provided informed consent, (2) aged between 17 and 24, (3) were enrolled in universities or colleges within China during the survey collection, (4) whose residence before college was in China and (5) who answered all questions and passed the consistency checks and logic verification. The final sample comprised 51,124 university and college students.

Ethical approval was obtained from the Institution Review Board of Tsinghua University.

### Exposures

Previous studies have found that parents' marital status was associated with adverse SRH outcomes, such as sexual abuse, HRSB, and unintended health outcomes [9–21], which have been discussed in the background part. Divorce is the most directly objective measure of parents’ marital status, and perceived parental marital quality is the subjective measure of parents’ marital status. Considering every single measurement may have limitations from the validity or reliability of measurement or from theoretical aspects, we included these two measurements in our study. Perceived parental marital quality as an emotional dimension of the home environment and divorce as significant family events were identified as two exposures in this study. The parental marital quality was assessed by the following question: “How would you rate the overall marital quality of your parents when you were growing up on a scale of 0 to 10?”. Although another study used a 5-point Likert scale [8], we chose a scale of 0 to 10 in order to capture more subtle differences. Higher scores reflect a better relationship. Standardized Z scores of perceived parental marital quality were computed and used in all regression analyses.

All participants were asked “Did you experience the following situations in your family?” with parents’ divorce as one choice. For those who experienced parents’ divorce, they were required to choose their parents’ divorce timing from early and middle childhood (around 12 years old or younger), adolescence (around 13–18 years old), and young adulthood (around 19 years old or older). Participants who experienced parents’ divorce were defined as children from “divorced family” (coded as 1) whereas those who did not were defined as from “intact family” (coded as 0).

### Outcomes

Based on the commonly used core concepts of SRH globally [30, 31], and previously mentioned SRH indicators measured in other studies examining the impact of parental marital status on young people’s SRH, we included sexual abuse, HRSB, and unintended health outcomes as the indicators of SRH in our study. The definition and variable selection of sexual abuse, high-risk sexual behaviors, and unintended health outcomes were based on current literature and China’s cultural background. Thirteen dichotomous variables (Yes/No) were generated to examine sexual experiences, HRSB, unintended health outcomes and sexual abuse. The respondents were asked to report whether they experienced any event separately. Sexual debut before the survey was used to indicate sexual intercourse experiences.

#### High-risk sexual behaviors

HRSB were evaluated by the first sexual intercourse with a non-intimate partner, casual sexual intercourse, multiple sexual partners and no contraceptive use in the last sexual intercourse [1, 13, 17, 19]. While first sexual intercourse with non-intimate and other casual sexual intercourse may not always have adverse consequences, these behaviors are more likely to pose higher SRH risks. This is the case primarily because these types of sexual behaviors tend to imply a lower sense of morality and responsibility in the Chinese context by contradicting loyalty to the partner or commitment to a relationship [32], and are more likely to be associated with unprotected sex [33, 34].

The first sexual partner of the respondents was dichotomized into “intimate” (defined as one’s boyfriend or girlfriend, wife or husband) and “non-intimate”. The accumulative numbers of sexual partners among sex-experienced respondents were classified into “one” and “multiple”. Information on casual sex (such as one-night stands) and no contraceptive use in the last sexual intercourse was obtained by asking “Have you ever had casual sex?” and “Did you use any contraceptives in the last sexual intercourse?”, with the answers “yes” or “no”.

#### Unintended health outcomes

Unintended health outcomes were assessed by unintended pregnancy (or pregnancy of the female partners in male respondents), induced abortion (or induced abortion of the female partners in male respondents), diagnosed STIs, and any genito-urinary system symptoms including urethral or vaginal discharge, painful urination, genital inflammation, genital ulcers, genital itching, genital herpes, and haematuria or vaginal bleeding [10, 11, 14]. Information on unintended health outcomes was obtained by asking, “Have you or your partner(s) ever had the following experiences?” with unintended health outcomes as options self-selected by participants. The experiences of sexual partners were only asked when measuring unintended pregnancy and induced abortion. Unintended health outcomes were categorized into “yes” and “no” as above.

#### Sexual abuse

Participants’ experiences of sexual abuse were measured on four fronts of progressive severity: forced to expose the genitals, forced to be kissed or fondled the genitals, forced to have oral intercourse, and forced to have penetrative vaginal or anal intercourse [35]. Respondents reported whether they had experienced these sexual abuse experiences and the results were recorded as “yes” and “no”.

### Covariates

Socio-demographic covariates included age, household economic status, home district, home region and parental education levels. All variables were coded as categorical variables. Participants were divided by age into three groups: 17–18 years, 19–20 years, and 21–24 years. Household economic status was assessed based on a 7-point scale according to self-appraisal, and higher scores indicate better family financial status. It was classified as “low” for those who chose 1–3 points, “medium” for 4 points and “high” for 5–7 points. The home district was defined as the place of residence before college enrollment, which was categorized into “urban” and “rural”. The home region was used to describe the residential area before college enrollment, which was classified into “eastern,” “middle” and “western”. Parental education levels were measured by the highest education levels of the mother and father separately. They were categorized into “no formal school” “elementary school,” “junior and senior high school” and “college and above”.

### Statistical Analysis

Although we use part of the data from NCSS-SRH which has a large sample size, we still conducted power calculations to determine whether the sample size for this study assures adequate power to detect statistical significance. We adopted commonly used assumptions, including a 5% margin of error, a power of 80%, and adding a 10% non-response rate to the minimum sample size estimated. Comparing all available studies, we found that the prevalence of sexual abuse among the general youth population in China is the relatively lowest prevalence compared to the prevalence of other outcomes chosen in this study. Since no study examined the prevalence of sexual abuse in adolescents from divorced families in China, we used the prevalence obtained from this study. As this figure is far below the Chinese average [36], it can ensure the sample size calculated will be large enough in Chinese context. We use the prevalence of 1.09% (from intact families) and 2.59% (from divorced families) of young people forced to have penetrative vaginal or anal intercourse which is the lowest prevalence in sexual abuse collected by this study, and the final sample size was calculated as 28,189, which was less than the sample size collected in this study. So, the final sample size of this study was large enough to provide sufficient power.

To improve the national representativeness of the sample, poststratification weights were added based on school locations, school types, grades, and sex according to the record of the Educational Statistics Yearbook of China 2018 [29]. This survey weight was added to all the statistical analyses of this study.

Descriptive statistics and regression analysis were performed. All demographic, socio-economic and SRH variables were presented in two groups: intact family and divorced family. Continuous variables were reported as the mean and standard deviation (SD) while categorical variables were described as percentage. We used multivariate logistic regression to assess the relationship of perceived parental marital quality and parents’ divorce with sexual intercourse, HRSB, unintended health outcomes and sexual abuse, stratified by sex. Since a statistically significant (P-value) result can be obtained if the sample is large enough, but the effect sizes are not directly affected by sample sizes [37]. We not only reported the P values, but also used the odds ratio (OR) and 95% confidence intervals (95% CIs) in our study as indicators of practical significance to show the size of the observed effect. Odds ratio (OR) and 95% confidence intervals (95% C.I.) were calculated to indicate the extent of association between the exposure and outcome variables. Considering the effect of a large sample size, P < 0.001 (two-sided) was considered as statistical significance. But we also reported the results when P < 0.05 and P < 0.01, given the fact that this is the level of statistical significance chosen for general studies. Age, household economic status, home district, home region and parental education levels were adjusted in all regression models. All data analyses were performed using Stata 16.

## Results

Participants’ characteristics, including demographic, social-economic and family status, were listed in Table 1. A total of 51,124 university or vocational college students were enrolled in this study, 10.72% of them came from divorced families. More than half of the participants aged between 19 and 20 years old. The scores of the perceived parental marital quality rated by students from divorced families were less than half that of students from intact families (3.22 vs. 7.44). The proportion of participants who had sexual experiences was higher in those from divorced families than their counterparts (30.27% vs. 19.95%). They also had a higher percentage of experiencing sexual abuse. 2.59% of students from the divorced families reported the forced penetrative vaginal or anal intercourse, which was almost twice of that in intact families (1.09%).

**Table 1:** Student characteristics from intact families and divorced families

Characteristics	Intact family N = 45,646 N (%)	Divorced family N = 5,478 N (%)
Age, years		
17–18	14,625 (32.04%)	1680 (30.67%)
19–20	22,964 (50.31%)	2801 (51.13%)
21–24	8057 (17.65%)	997 (18.20%)
Mean (SD)	19.29 (1.35)	19.32 (1.37)
Sex		
Female	29,807 (65.30%)	3846 (70.21%)
Male	15,839 (34.70%)	1632 (29.79%)
Home district		
Urban	35,143 (76.99%)	4736 (86.45%)
Rural	10,503 (23.01%)	742 (13.55%)
Home region		
Eastern	20,837 (45.65%)	2268 (41.40%)
Middle	13,096 (28.69%)	1490 (27.20%)
Western	11,713 (25.66%)	1720 (31.40%)
Household economic status		
Low	11,147 (24.42%)	1805 (32.95%)
Medium	17,432 (38.19%)	2032 (37.09%)
High	17,067 (37.39%)	1641 (29.96%)
Paternal education level		
No formal school	2200 (4.82%)	454 (8.29%)
Elementary school	7536 (16.51%)	769 (14.04%)
Junior and senior high school	27,351 (59.92%)	3067 (55.98%)
College and above	8559 (18.75%)	1188 (21.69%)
Maternal education level		
No formal school	4400 (9.64%)	507 (9.26%)
Elementary school	10,891 (23.86%)	932 (17.00%)
Junior and senior high school	23,677 (51.87%)	2975 (54.31%)
College and above	6678 (14.63%)	1064 (19.43%)
Perceived parental marital quality	7.44 (2.15)	3.22 (2.62)
Mean (SD)		
Ever had sexual intercourse	9106 (19.95%)	1658 (30.27%)
Sexual abuse		
Forced to expose the genitals	1876 (4.11%)	356 (6.50%)
Forced to be kissed or fondled the genitals	4241 (9.29%)	840 (15.33%)
Forced to have oral intercourse	653 (1.43%)	173 (3.16%)
Forced to have penetrative vaginal or anal intercourse	498 (1.09%)	142 (2.59%)

Table 2 focuses on the association between sexual behaviors, SRH outcomes and family marital status. Based on sex-experienced responses, students from divorced families were more likely to have HRSB and unintended health outcomes than intact families. In particular, a higher percentage of students in divorced families had their first sexual intercourse with a non-intimate (13.75% vs. 10.61%), and they reported more frequent casual sexual intercourse (22.44% vs. 15.81%). 5.07% of students from divorced families had pregnancy or experienced partner pregnancy, but only 4.12% in intact families. A similar pattern can be observed in students who have had a sexually transmitted infection (4.16% vs. 2.14%).

**Table 2:** Sexual behaviors from intact families and divorced families of sex-experienced respondents

Characteristics	Intact family N = 9107 N (%)	Divorced family N = 1658 N (%)	P value
High-risk sexual behaviors			
Had first sexual intercourse with non-intimate	966 (10.61%)	228 (13.75%)	p = 0.001
Ever had casual sexual intercourse	1440 (15.81%)	372 (22.44%)	p < 0.001
Had multiple sexual partners	3716 (40.80%)	852 (51.39%)	p < 0.001
No contraceptive use at last intercourse	372 (4.09%)	93 (5.62%)	p = 0.021
Unintended health outcomes			
Had pregnancy/Got partner pregnant	375 (4.12%)	84 (5.07%)	p = 0.064
Had induced abortion/Partner had induced abortion	339 (3.72%)	76 (4.58%)	p = 0.084
Had genito-urinary system symptom	2758 (30.28%)	595 (35.89%)	p < 0.001
Had sexually transmitted infection	195 (2.14%)	69 (4.16%)	p < 0.001

Table 3 presents the correlation between perceived parental marital quality and youths’ SRH. A better perceived parental marital quality prevented the occurrence of sexual intercourse, sexual abuse, HRSB, and unintended health outcomes. The increase in the standardized parental marital quality score was associated with a lower probability of sexual intercourse (male: OR: 0.83, 95% CI: 0.80–0.86; female: OR: 0.73, 95% CI: 0.70–0.75) and sexual abuse such as forced penetrative vaginal or anal intercourse (male: OR: 0.73, 95% CI: 0.64–0.83; female: OR:0.71, 95% CI: 0.65–0.77). Better perceived parental marital quality was associated with lower odds of HRSB such as casual sexual intercourse (male: OR: 0.78, 95% CI: 0.73–0.83; female: OR: 0.77, 95% CI: 0.72–0.83), and unintended health outcomes including STI (male: OR: 0.79, 95% CI: 0.70–0.89; female: OR: 0.82, 95% CI: 0.73–0.91). There was no significant association between parental marital quality and contraceptive use at the last sexual intercourse in both males and females. The association between pregnancy, induced abortion experiences and perceived parental marital quality were only observed in females.

**Table 3:** The odds ratios of SRH associated with every 1-SD change in perceived parental marital quality

Variables	Male	Female
All respondents	N = 17,470	N = 33,654
Sexual intercourse		
Ever had sexual intercourse	0.83 (0.80–0.86)***	0.73 (0.70–0.75)***
Sexual abuse		
Forced to expose the genitals	0.81 (0.76–0.86)***	0.72 (0.68–0.76)***
Forced to be kissed or fondled the genitals	0.79 (0.75–0.84)***	0.74 (0.71–0.76)***
Forced to have oral intercourse	0.75 (0.67–0.84)***	0.71 (0.66–0.77)***
Forced to have penetrative vaginal or anal intercourse	0.73 (0.64–0.83)***	0.71 (0.65–0.77)***
Sex-experienced respondents	N = 4630	N = 6135
High-risk sexual behaviors		
Had first sexual intercourse with non-intimate	0.80 (0.74–0.86)***	0.83 (0.76–0.91)***
Ever had casual sexual intercourse	0.78 (0.73–0.83)***	0.77 (0.72–0.83)***
Had multiple sexual partners	0.86 (0.81–0.91)***	0.84 (0.80–0.88)***
No contraceptive use at last intercourse	0.96 (0.84–1.10)	0.92 (0.81–1.05)
Unintended health outcomes		
Had pregnancy/Got partner pregnant	1.00 (0.87–1.15)	0.87 (0.77–0.98)*
Had induced abortion/Partner had induced abortion	0.96 (0.83–1.11)	0.86 (0.76–0.98)*
Had genito-urinary system symptom	0.84 (0.80–0.88)***	0.82 (0.80–0.84)***
Had sexually transmitted infection	0.79 (0.70–0.89)***	0.82 (0.73–0.91)***

Adjusted for age, household economic status, home district, home region and parental education levels. *p < 0.05, **p < 0.01, ***p < 0.001

Table 4 shows the associations between the timing of parents’ divorce and youths’ SRH. We found a positive association between parents’ divorce time and sexual intercourse in both males and females, with a higher risk of sexual intercourse in the divorced group especially in early and middle childhood (male: OR: 1.60, 95% CI: 1.40–1.84; female: OR: 1.85, 95% CI: 1.67–2.04). Unintended health outcomes were increased in females when the divorce occurred in their early and middle childhood, such as pregnancy (OR: 1.46, 95% CI: 1.00–2.12), and induced abortion (OR: 1.49, 95% CI: 1.02–2.17). However, there were no associations between the two in males, or in females whose parents divorced in their adolescence or later. Additionally, parental divorce was associated with a higher likelihood of sexual abuse. For example, those whose parents divorced in their adolescence or later were more likely to have had forced penetrative intercourse (male: OR: 3.41, 95% CI: 2.06–5.62; female: OR: 2.53, 95% CI: 1.83–3.49). A higher occurrence risk of HRSB was observed mainly in those whose parents divorced during adolescence or later. The odds ratios for ever having casual sexual intercourse in participants whose parents divorced during adolescence or young adulthood were 1.84 (95% CI: 1.35–2.51) in males and 1.68 (95% CI: 1.25–2.18) in females.

**Table 4:** The odds ratios of SRH associated with parents' marital status

Variables	Male			Female		
	Intact family	Parents divorced in early and middle childhood	Parents divorced during adolescence or young adulthood	Intact family	Parents divorced in early and middle childhood	Parents divorced during adolescence or young adulthood
All respondents	N = 15,838	N = 1055	N = 577	N = 29,808	N = 2483	N = 1363
Sexual intercourse						
Ever had sexual intercourse	1	1.60 (1.40–1.84)***	1.45 (1.21–1.75)***	1	1.85 (1.67–2.04)***	1.70 (1.49–1.94)***
Sexual abuse						
Forced to expose the genitals	1	1.09 (0.84–1.42)	1.63 (1.21–2.19)**	1	1.55 (1.29–1.87)***	1.88 (1.51–2.35)***
Forced to be kissed or fondled the genitals	1	1.67 (1.36–2.05)***	1.57 (1.19–2.08)**	1	1.59 (1.42–1.78)***	1.49 (1.28–1.73)***
Forced to have oral intercourse	1	2.13 (1.47–3.09)***	1.79 (1.05–3.06)*	1	1.88 (1.45–2.42)***	2.19 (1.61–2.97)***
Forced to have penetrative vaginal or anal intercourse	1	2.13 (1.35–3.34)**	3.41 (2.06–5.62)***	1	1.84 (1.38–2.46)***	2.53 (1.83–3.49)***
Sex-experienced respondents	N = 4049	N = 379	N = 202	N = 5058	N = 698	N = 379
High-risk sexual behaviors						
Had first sexual intercourse with non-intimate	1	1.40 (1.07–1.84)*	1.63 (1.15–2.31)**	1	1.23 (0.92–1.64)	1.66 (1.19–2.32)**
Ever had casual sexual intercourse	1	1.68 (1.33–2.14)***	1.84 (1.35–2.51)***	1	1.55 (1.24–1.93)***	1.68 (1.25–2.18)***
Had multiple sexual partners	1	1.65 (1.33–2.05)***	2.18 (1.62–2.94)***	1	1.48 (1.25–1.74)***	1.45 (1.17–1.79)**
No contraceptive use at last intercourse	1	1.40 (0.90–2.17)	1.85 (1.08–3.18)*	1	1.40 (0.95–2.06)	1.08 (0.62–1.90)
Unintended health outcomes						
Had pregnancy/Got partner pregnant	1	0.91 (0.54–1.55)	1.52 (0.84–2.73)	1	1.46 (1.00–2.12)*	1.30 (0.78–2.16)
Had induced abortion/Partner had induced abortion	1	1.05 (0.62–1.79)	1.43 (0.76–2.70)	1	1.49 (1.02–2.17)*	0.97 (0.64–1.74)
Had genito-urinary system symptom	1	1.13 (0.95–1.35)	1.13 (0.89–1.43)	1	1.27 (1.15–1.39)***	1.27 (1.13–1.44)***
Had sexually transmitted infection	1	1.62 (1.04–2.53)*	2.77 (1.69–4.54)***	1	1.52 (1.05–2.20)*	2.34 (1.57–3.49)***

Adjusted for age, household economic status, home district, home region and parental education levels. *p < 0.05, **p < 0.01, ***p < 0.001

## Discussion

This study found that poor parental marital relationships and divorce were associated with sexual intercourse, sexual abuse, HRSB, and unintended health outcomes in Chinese college and university students. The associations differed slightly across females and males, with a stronger impact on females. Moreover, the risk of sexual abuse, HRSB, and unintended health outcomes varied across the time of parental divorce.

The results illustrated that unhealthy parental marital relationships and parents’ divorce contribute to higher possibilities of HRSB, sexual abuse, and unintended health outcomes, which is consistent with previous studies. For example, studies conducted in other countries also observed similar associations between better perceived parental marital quality and reduced odds of having HRSB in married-parent families [7, 8, 20]. The reasons behind such relationships included that lack of parents’ love, support, and supervision due to divorce are risk factors for HRSB in adolescents, which may be the same across cultures [38–40]. On the one hand, parental divorce may prevent adolescents from receiving support from one parent who may move out of the household, which may increase the risk of children being more exposed to risky sexual behaviors and having poorer SRH outcomes. On the other hand, when there is marital conflict, parents may be less collaborative in supervising their children and provide less family-based sexual education, which may lead to indulging the child's potential for HRSB. In addition, consistent with our findings, college students from divorced families were more likely to have an unintended pregnancy or make their sex partners pregnant [41]. Although some evidence indicated that female college students whose parents divorced in early and middle childhood were more likely to have had an induced abortion [42], we did not observe the association in the current study.

We encountered sex disparities in unintended health outcomes related to parental marital relationships and divorce. We found that low parental marital quality increased the risk of pregnancy and induced abortion experiences for Chinese adolescents, which were only observed in females. Consistent with our findings, another study in 952 adolescents in the United States (US), also observed a similar relationship between family support and sexual behaviors only in females [43]. In addition, a systematic review discussed the risk factors for early sexual debut and coerced sex. They concluded that unhealthy family relationships, such as divorce and the absence of parental support, became more common and debilitating for girls [44]. This could be partially attributable to the fact that females are more sensitive and require more emotional support from parents in childhood and adolescence than males [45]. Moreover, girls may be more likely to feel stressed because of their sensitivity [46], when in divorce families or their parental marital relationships being worse. Parental divorce or worse parental marital relationships, as stressful events for children, may thus influence more girls than boys. In future studies, we may consider the sensitivity to stress as a moderating factor to further investigate the gender difference in these relationships.

In the present study, several HRSB were significantly associated with divorce timing, consistent with a previous study in the US [47]. Interestingly, HRSB may be partially caused by parental neglect during childhood. Because lacking parent accompaniment influences individual development, and results in delays in psychological processes, such as reliance and emotions [48]. We confirmed that adolescents and young adults with divorced parents were more likely to engage in risky sexual activity, which may raise the risk of unintended health outcomes. This suggests that the accompanying stressors in the families are more likely to increase the HRSBs during the transitional period to adulthood. There are several factors that may contribute to students' HRSBs related to divorce. Children with divorced parents may have less sense of bond with their parents due to decreased contact [11, 49]. Besides, unhealthy marriage contains more arguments and stress, which may lead to financial hardships and less attention for children [50]. Moreover, divorced parents are more likely to accept or be indifferent to their children’s sexual behaviors such as early sexual intercourse [26, 38]. Such neglect of the child may hinder them from knowing what are HRSBs or how to prevent HRSB from happening again, compared with those from intact families who can get support and sex education from parents. On the other hand, when individuals reach their teen years, the anger about a divorce may increase their engagement in premature romantic relationships and HRSB [51]. The emotional stress from parents’ divorce may reduce the ability to maintain a long and stable relationship [52], which may result in an increased number of sexual partners.

In this study, we not only considered the effect of family structure on children's SRH, but also further considered the effect of the relationship between parents. We found that both family structure, as indicated by parental divorce, and parental marital relationships were associated with poor SRH. A study from Spain found that adolescents from a low-marital-quality family with married parents are more likely to engage in risky sexual behaviors than those from low-conflict relationships with divorced parents [8]. In future research, we can also compare which has a greater impact on young people in the Chinese context: parental relationships or family structure intactness. Besides, unlike western countries, such as the US [53], in the cultural context of China, people generally choose to have and raise children during marriage instead of in cohabiting unions as in the past [54]. So, this study did not consider the family stability and parental marital quality of cohabiting families, and their impact on adolescent SRH. In China, convergence with trends in the family behaviors of Western societies is being gradually observed, namely a rapid increase in cohabitation [55]. Therefore, in future studies, it is recommended to consider not only the impact of parental marital relationships on children's SRH but also to include cohabitation as an influencing factor.

### Strengths and limitations

We conduct a comprehensive study to explore the association between parents’ marital status and quality and SRH in Chinese youth, which helps expand the literature to understand the sexual risk-taking and adverse health outcomes of a more diverse population. We collected large-scale samples with proper geographic distribution, improving credibility and generalizability across China. We strengthened the attention to the importance of a positive parental marital relationship and its long-term effects on children’s SRH. Our social media-based approach could maximize respondents’ privacy protection and avoid socially desirable responses. Finally, the outcome variables included in this study ranged from sexual intercourse to HRSB, unintended health outcomes and sexual abuse. These developed a comprehensive understanding of family/marriage influences on sexuality.

In addition to its strengths, this study had several limitations. Since this is an online survey, all information was self-reported, and these data were susceptible to recall bias. Since social attitudes toward sex are traditionally conservative and ashamed in China, it is possible that the self-reported prevalence of HRSB or sexual abuse is lower than the actual cases. Moreover, as the data is from a study comprehensively measured adolescent SRH in China, it collected a large amount of information on SRH attitude, behavior and outcomes. But it used relatively simple measures for sociodemographic questions in order to prevent too lengthy questionnaires and respondent burnout, and to improve the quality and response rate of online questionnaires. For example, parental marital quality was measured by a single question based on participants’ self-reported data, and the validity and reliability of the measurement may not be as high as in other studies that were specifically designed to research parental marital quality. However, the 5-point Likert scale measurement used in other studies was extended by us to a 0–10 scale, which is more refined. We may have obtained data with higher variability, but the validity and reliability of sociodemographic questions measurement should still be improved in further studies. Future research could also take some other approaches to help collect more accurate information, such as linking this database to the Chinese census database. In addition, the sexual behaviors of young adults not enrolled in higher education were not addressed here. Furthermore, this cross-sectional study was unable to measure the potential dynamic of parental marital quality over time or whether a change in parental marital status has influenced marital quality and led to subsequent changes in SRH in youth. Future research is encouraged to further explore the potential impact of interpersonal violence, parent-parent relationship or parent–child relationship on ASRH in youth.

## Conclusions

We demonstrated that poor parental marital quality and divorce were positively associated with sexual intercourse, HRSBs, unintended health outcomes and sexual abuse in college students. Some associations were female-specific. The findings provide implications for implementing intervention programs to help unharmonious families and divorced families, especially for female children. School-based and family-based sexuality education is required for children being involved in family discord, conflict, and breakdown, and divorced parents should consider providing more attention and guidance to their children in daily life.

## Data Availability

The data are not publicly available due to conditions on participant consent and other ethical restrictions, as they contain information that could identify and therefore compromise the privacy of research participants.

## Abbreviations

HRSB: High-risk sexual behaviors
STI: Sexually transmitted infections
SRH: Sexual and reproductive health
NCSS-SRH: National College Student Survey on Sexual and Reproductive Health
CFPA: China Family Planning Association
CYN: China Youth Network
HEI: Higher education institution
SD: Standard deviation
OR: Odds ratio
CI: Confidence Interval
US: The United States

## References

[1] Fentahun N, Mamo A (2014). Risky sexual behaviors and associated factors among male and female students in Jimma Zone preparatory schools, South West Ethiopia: comparative study. Ethiop J Health Sci.

[2] Yu J (2012). Teenage sexual attitudes and behaviour in China: a literature review. Health Soc Care Community.

[3] Zhao R, Zhang L, Fu X, Su C, Zhang Y (2019). Sexual and reproductive health related knowledge, attitude and behavior among senior high school and college students in 11 provinces and municipalities of China. Chin J Public Health.

[4] Repetti RL, Taylor SE, Seeman TE (2002). Risky families: family social environments and the mental and physical health of offspring. Psychol Bull.

[5] Cavanagh SE, Crissey SR, Raley RK (2008). Family structure history and adolescent romance. J Marriage Fam.

[6] Donahue KL, D'Onofrio BM, Bates JE, Lansford JE, Dodge KA, Pettit GS (2010). Early exposure to parents' relationship instability: implications for sexual behavior and depression in adolescence. J Adolesc Health..

[7] Orgilés M (2012). Sexual behavior in Spanish adolescents of divorced parents. Psicothema.

[8] Orgilés M, Carratalá E, Espada JP (2015). Perceived quality of the parental relationship and divorce effects on sexual behaviour in Spanish adolescents. Psychol Health Med.

[9] Santelli JS, Lowry R, Brener ND, Robin L (2000). The association of sexual behaviors with socioeconomic status, family structure, and race/ethnicity among US adolescents. Am J Public Health.

[10] Magnani RJ, Seiber EE, Gutierrez EZ, Vereau D (2001). Correlates of sexual activity and condom use among secondary-school students in urban Peru. Stud Fam Plann.

[11] Bakken RJ, Winter M (2002). Family characteristics and sexual risk behaviors among black men in the United States. Perspect Sex Reprod Health.

[12] Langille DB, Curtis L, Hughes J, Murphy GT (2003). Association of socio–economic factors with health risk behaviours among high school students in rural Nova Scotia. Can J Public Health.

[13] Vukovic DS, Bjegovic VM (2007). Brief report: risky sexual behavior of adolescents in Belgrade: association with socioeconomic status and family structure. J Adolesc.

[14] Peres CA, Rutherford G, Borges G, Galano E, Hudes ES, Hearst N (2008). Family structure and adolescent sexual behavior in a poor area of Sao Paulo. Brazil J Adolesc Health.

[15] Lansford JE (2009). Parental divorce and children's adjustment. Perspect Psychol Sci.

[16] Upadhyay UD, Hindin MJ (2007). The influence of parents' marital relationship and women's status on children's age at first sex in Cebu, Philippines. Stud Family Plan.

[17] Cleveland HH, Gilson M (2004). The effects of neighborhood proportion of single–parent families and mother–adolescent relationships on adolescents' number of sexual partners. J Youth Adolesc.

[18] D'Onofrio BM, Turkheimer E, Emery RE, Slutske WS, Heath AC, Madden PA, Martin NG (2006). A genetically informed study of the processes underlying the association between parental marital instability and offspring adjustment. Dev Psychol.

[19] Zimmer-Gembeck MJ, Helfand M (2008). Ten years of longitudinal research on U.S. adolescent sexual behavior: developmental correlates of sexual intercourse, and the importance of age, gender and ethnic background. Dev Rev.

[20] Kaye K, Moore KA, Hair EC, Hadley AM, Day RD, Orthner DK (2009). Parent marital quality and the parent–adolescent relationship: effects on sexual activity among adolescents and youth. Marriage Fam Rev.

[21] Wight D, Williamson L, Henderson M (2006). Parental influences on young people's sexual behaviour: a longitudinal analysis. J Adolesc.

[22] United Nations (2011). Demographic Yearbook. United Nations: New York.

[23] Ministry of Civil Affairs. Statistical Communiqué of the People's Republic of China on the 2020 Civil Affairs Development. http://images3.mca.gov.cn/www2017/file/202109/1631265147970.pdf. Accessed 7 Sept 2022.

[24] Zhang XB, Wang JW (2021). Analysis of the change of divorce level of Chinese youth since the reform and opening up. China Youth Study.

[25] Chen M, Rizzi EL, Yip PS (2021). Divorce trends in China across time and space: an update. Asian Popul Stud.

[26] Jeynes WH (2001). The effects of recent parental divorce on their children's sexual attitudes and behavior. J Divorce Remarriage.

[27] Quinlan RJ (2003). Father absence, parental care, and female reproductive development. Evol Hum Behav.

[28] Everett C (2014). Divorce and the next generation: effects on young adults' patterns of intimacy and expectations for marriage.

[29] Department of Development and Planning, Ministry of Education of the People's Republic of China. Educational Statistics Yearbook of China 2018; China Statistics Press: Beijing, China, 2019. [**In Chinese**]

[30] Hawkes S (2014). Sexual health: a post-2015 palimpsest in global health?. Lancet Glob Health.

[31] World Health Organization. Measuring sexual health: Conceptual and practical considerations and related indicators; Switzerland: Geneva, 2010. https://apps.who.int/iris/handle/10665/70434. Accessed 7 Sept 2022.

[32] Zhu XS, Zeng XL, Zhou H, Lin DH (2012). Sexual attitude of college students and its influencing factors. Chinese J Clin Psychol..

[33] Yang S, Jike C, Pei R, Liu D, Yu G, Wang J (2021). Perceptions of social norms played an important role in the occurrence of casual sex among Yi minority residents in China: a population-based study. AIDS Care.

[34] Liu B, He B, Zhu J (2019). Motivation and risk: social work analysis and response of college students' online casual sex. Youth Adolesc Stud..

[35] Negriff S, Schneiderman JU, Smith C, Schreyer JK, Trickett PK (2014). Characterizing the sexual abuse experiences of young adolescents. Child Abuse Negl..

[36] Ma Y (2018). Prevalence of childhood sexual abuse in China: a meta-analysis. J Child Sex Abus.

[37] Lantz B (2013). The large sample size fallacy. Scand J Caring Sci.

[38] Davis EC, Friel LV (2001). Adolescent sexuality: disentangling the effects of family structure and family context. J Marriage Fam.

[39] Ellis BJ, Bates JE, Dodge KA, Fergusson DM, John Horwood L, Pettit GS (2003). Does father absence place daughters at special risk for early sexual activity and teenage pregnancy?. Child Dev.

[40] Ream GL, Savin-Williams RC (2005). Reciprocal associations between adolescent sexual activity and quality of youth–parent interactions. J Fam Psychol.

[41] Browder SE. Family influences on risky sexual behaviors, pregnancy, and abortion decisions in Swiss adolescents. Doctoral Dissertation, Auburn University, Alabama, 2008.

[42] Bleil ME, Adler NE, Pasch LA, Sternfeld B, Reijo-Pera RA, Cedars MI (2011). Adverse childhood experiences and repeat induced abortion. Am J Obstet Gynecol..

[43] Mmari K, Kalamar AM, Brahmbhatt H, Venables E (2016). The influence of the family on adolescent sexual experience: a comparison between Baltimore and Johannesburg. PLoS ONE.

[44] Lee RLT, Yuen LA, Hung TTM, Sobel H (2018). A systematic review on identifying risk factors associated with early sexual debut and coerced sex among adolescents and young people in communities. J Clin Nurs.

[45] Kincaid C, Jones DJ, Sterrett E, McKee L (2012). A review of parenting and adolescent sexual behavior: the moderating role of gender. Clin Psychol Rev.

[46] Fischer AH, Kret ME, Broekens J (2018). Gender differences in emotion perception and self-reported emotional intelligence: A test of the emotion sensitivity hypothesis. PLoS ONE.

[47] Anderson KG (2017). Adverse childhood environment: Relationship with sexual risk behaviors and marital status in a large American sample. Evol Psychol.

[48] Kogan SM, Cho J, Oshri A (2016). The influence of childhood adversity on rural black men's sexual risk behavior. Ann Behav Med.

[49] Amato PR, Keith B (1991). Parental divorce and the well–being of children: a meta–analysis. Psychol Bull.

[50] Grych JH, Fincham FD (1990). Marital conflict and children's adjustment: A cognitive–contextual framework. Psychol Bull.

[51] Moreno C, Sánchez-Queija I, Muñoz-Tinoco V, de Matos MG, Dallago L, Ter Bogt T (2009). Cross–national associations between parent and peer communication and psychological complaints. Int J Public Health.

[52] Jónsson FH, Njarðvik U, Ólafsdóttir G, Grétarsson S (2000). Parental divorce: Long-term effects on mental health, family relations and adult sexual behavior. Scand J Psychol.

[53] Manning WD (2015). Cohabitation and child wellbeing. Future Child.

[54] Yu J, Xie Y (2021). Recent trends in the Chinese family: national estimates from 1990 to 2010. Acta Pharmacol Sin.

[55] Yu J, Xie Y (2015). Cohabitation in China: trends and determinants. Popul Dev Rev.

